# Ectodermal disturbance in development shared by anorexia and schizophrenia may reflect neurodevelopmental abnormalities

**DOI:** 10.1002/brb3.2281

**Published:** 2021-09-12

**Authors:** Barbara Remberk, Piotr Niwiński, Ewa Brzóska‐Konkol, Anna Borowska, Anna Papasz‐Siemieniuk, Joanna Brągoszewska, Anna Katarzyna Bażyńska, Łukasz Szostakiewicz, Anna Herman

**Affiliations:** ^1^ Institute of Psychiatry and Neurology Warsaw Poland; ^2^ Psychological and Pedagogical Counselling Centre no 7 Warsaw Poland; ^3^ Medical University of Warsaw Warsaw Poland

**Keywords:** adolescent, anorexia, minor physical abnormalities, neurodevelopment, schizophrenia

## Abstract

Minor physical abnormalities (MPA) are subtle dysmorphic features of bodily structures that have little or no impact on function. Most MPA develop during the first gestational trimester and are considered as important indicators of neuroectodermal deficiencies emerging during early brain development. A higher frequency of MPA was confirmed in schizophrenia patients and their relatives, when compared to controls. These findings are consistent with the neurodevelopmental model of schizophrenia. A neurodevelopmental component amongst other risk factors has also been recently proposed for anorexia nervosa (AN). The current study aimed to assess MPA frequency in adolescent inpatients with either schizophrenia spectrum disorders (SSD) or AN as compared to healthy controls (HC). The Waldrop Scale was used for assessing MPA. The mean MPA total score and mean head subscore was significantly higher in both test groups than in HC. There were no statistically significant differences between SSD and AN groups. The MPA profile (not frequency) was similar in all three groups. This finding is consistent both with widely acknowledged neurodevelopmental schizophrenia hypothesis as well as with more recent neurodevelopmental model of AN. Nevertheless, the findings should not be overgeneralized and further studies are warranted.

## Graphical abstract

Minor physical abnormalities (MPA) may indicate neuroectodermal developmental deficiencies. MPA frequency was assessed in adolescents with schizophrenia spectrum disorders (SSD) anorexia nervosa (AN) and healthy controls (HC). The mean MPA score was significantly higher in both test groups than in HC. The results are consistent with neurodevelopmental schizophrenia hypothesis and indicate neurodevelopmental etiological component in anorexia nervosa.

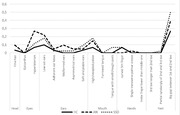

## 1 INTRODUCTION

Minor physical anomalies (MPA) consist of subtle dysmorphic features that have minor, or are devoid of functional consequences. Because most MPA develop during the first gestational trimester and are ectoderm‐associated (skin), they are also taken as valuable indicators of neuroectodermal disorders whenever arising during the early development of the brain (Franco et al., 2010; Tényi et al., 2009).

Studies have confirmed higher rates of MPA in patients with schizophrenia compared with controls (John et al., 2008; Lloyd et al., 2008; Torrey et al., 2012).This is also observed amongst different ethnic groups (Dean et al., 2007; Koen et al., 2006). Risk and protective factors of psychosis have been systematically reviewed from 683 publications (1965–2017) in an umbrella review (Radua et al., 2018), where MPA were found to be one out of six highly suggestive factors associated with psychosis. Moreover, high rates of mouth area‐MPA were demonstrated in diagnosed cases of schizophrenia that were particularly associated with treatment‐resistance (Lin et al., 2015), as well as propensities for tardive dyskinesia (Lohr & Flynn, 1993) and homicidal behavior (Tényi et al., 2015). High rates of MPA were also associated with a family history of schizophrenia, obstetric complications, lower number of siblings, later position in birth order and male gender (O'Callaghan et al., 1991). Anomalies in the craniofacial regions were also more common in patients’ unaffected relatives than in controls (Hajnal et al., 2016; Wang et al., 2016). Early onset schizophrenia (EOS) was associated with more MPA in different subsamples than in adult‐onset schizophrenia (AOS) (Green et al., 1987; Hata et al., 2003) and was in this sample linked with brain morphology abnormalities (Hata et al., 2003); in keeping with the notion suggesting a greater biological burden in EAO compared to AOS.

A Danish cohort study (Golembo‐Smith et al., 2012; Schiffman et al., 2002) showed that the presence of MPA present in premorbid phase, (along with other markers of neurodevelopment disturbances), predicted the development of schizophrenia spectrum disorder (SSD). A study by Blanchard et al. (2010) showed high MPA scores in a population‐based sample which were associated with social anhedonia together with schizoid and schizotypal personality dimensions. Schizotypal symptoms also correlated with MPA in those adolescents having parents with schizophrenia (Hans et al., 2009). Adolescents that fulfilled the DSM‐IV criteria for schizotypal personality disorders, were observed to have more MPA than either controls or those subjects with other personality disorders (Weinstein et al., 1999).

MPA are however not just specific to schizophrenia. They have also been found in children suffering from other conditions, such as autism spectrum disorder (Campbell et al., 1978; Myers et al., 2017), attention deficit and hyperactivity disorder (Myers et al., 2017), hyperkinetic behavior (Gualtieri et al., 1982), intellectual disability (Myers et al., 2017; Ulovec et al., 2002) and hearing or visual impairment (Ulovec et al., 2012). The majority of these conditions fall within the ICD‐11 and DSM‐5 category of “Neurodevelopmental disorders.”

Certain MPAs were found to coexist with substance abuse and early sexual practice in a study on an adolescent cohort taken from a low economic background, however this study had many limitations (Tsai et al., 2018).

In adults, MPA were observed in bipolar patients, (both bipolar I and bipolar II) (Berecz et al., 2017; Lloyd et al., 2008), where rates of MPA in such patients were found to be intermediate between schizophrenia subjects and controls (Akabaliev et al., 2014). MPAs were also observed in female patients with recurrent depression; similar rates in those psychotic and non‐psychotic (Culav‐Sumic et al., 2010). Moreover, despite there being no difference in MPA scores between alcohol dependent patients and controls, the distribution of scores was bimodal; suggesting group heterogeneity (Gualtieri et al., 1982).

Schizophrenia is a disorder with a complex etiology including biological and environmental factors. Numerous alterations in brain morphology, function, cytoarchitecture, a confirmed role of pre‐ and perinatal factors along with genetic findings, confirm the neurodevelopmental model of schizophrenia (Birnbaum & Weinberger 2017; Rund, 2018). At the structural level, schizophrenia is associated with the following anatomical correlates: The medial prefrontal cortex, the upper temporal furrow, the fusiform gyrus, the amygdala, and the abdominal‐medial prefrontal cortex. Furthermore, brain imaging has demonstrated that schizophrenia is linked to irregularities in brain structure such as reduced total brain volume, enlarged ventricles and furrows, abnormalities in the temporal lobe and reductions of the hippocampus, amygdala and other areas (Keshavan et al., 2020). EOS is considered a more severe form of the disorder that bears a substantial endogenous etiological component (Hilker et al., 2017).

Initially, AN was suggested to be associated with psychological factors such as a dysfunctional family as well as social and cultural burdens (Keel & Klump 2003). Pharmacological agents are considered ineffective in these circumstances and psychotherapy is the recommended approach (NICE, 2017). Nevertheless, the role of biological factors is now acknowledged (Keel & Klump 2003; Phillipou et al., 2014).

A neurodevelopmental component had been suggested by Connan et al. (2003) and is now being developed. A number of prenatal risk factors have been found: Abnormal testosterone balance, maternal infections like chickenpox or rubella, and low maternal vitamin D levels. Other risk factors include prenatal complications, low weight for gestational age premature birth, high parental ages at childbirth, caesarean section, and congenital malformations of the mouth and digestive system (Larsen et al., 2020; Raevuori et al., 2014). Obstetric complications increase the risk of AN, but not bulimia nervosa (Raevuori et al., 2014). Other supporting studies have confirmed a significant heritability component in eating disorders. The heritability rate of AN accounts for 50–80% cases, which is comparable to heritability rates of those disorders with a confirmed neurodevelopmental component such as schizophrenia (Bulik et al., 2019; Larsen et al., 2020).

Abnormalities in brain structure and function have been observed in patients with AN. Some appear to be reversible after weight restoration (Cascino et al., 2020; Hay, 2011).A decreased grey matter volume in the fronto‐parietal area was inversely correlated with illness duration and psychopathology severity, but not with body mass index (Bär et al., 2015).Highly increased cortical folding, found in both the right dorsolateral prefrontal cortex and the right visual cortex, has been observed in patients with AN, but is independent of symptom severity (Schultz et al., 2017). Other studies have found the local folding index (LFI) in the postcentral area to be associated with acute AN symptoms (Miles et al., 2018), whilst decreased LFI in the frontal cortex was observed irrespective of the illness phase.

Functional disturbances of central nervous system (CNS) are also observed in AN. Evidence suggests that body image processing includes alterations in the insula reaction to olfactory stimuli (Wagner et al., 2008), changes in right parietal lobe function and interactions between inferior parietal lobule, insula, and subthalamus (Case et al., 2012; Grunwald et al., 2001). An increased 5HTIA receptor density in the fronto‐temporo‐parietal area was found at different illness stages. Furthermore, 5‐HT transporter binding in numerous brain areas had been changed. Studies focused on regional blood flow, suggest the importance of temporal lobes and connectivity dysfunctions between temporal areas and cingulate cortex in eating disorders (Gianni et al. 2020). Dysregulatedneurosecretion of serotonin is observed in AN, both in its acute phase, (during progressive physical and mental cachexia), and when weight is being restored at psychiatric remission. Not only does the neurosecretory system significantly affect mood and well‐being, but it also modulates appetite, motor activity, impulse control and obsessive‐compulsive behavior. A complex model proposed by Kaye et al. (2011), describes links between higher order cortical modulation associated with food intake and satiety stimuli processing with dysfunctional sensory interpretation of somatic, autonomic and cenesthetic stimuli.

Further genetic studies support a neurodevelopmental component to AN aetiology (Bulik et al., 2019). Alterations were found in those genes encoding contactins, a subgroup of immunoglobulin cell adhesion molecules involved in neurodevelopment. This was described in cases of AN, alcohol abuse disorder and other disorders of neurodevelopmental etiology (Oguro‐Ando et al., 2017), where a Genome Wide Association Study revealed an important positive genetic correlation between AN and other disorders, including schizophrenia (Bulik‐Sullivan et al., 2015).

The role of postnatal adversities in the development of AN was also confirmed in those affecting CNS development, associated with attachment disorder or other traumatic experiences (Shore, 2009).

The following summary can be made from these quoted studies:

1. A neurodevelopmental component forms part of anorexia's etiology.
2. There exist some as yet ill‐defined associations between anorexia and schizophrenia.

The scientific utility of MPA examination, as a mode of assessment, has been confirmed in schizophrenia studies. This method has however not been previously used in anorexia studies,

The current study has aimed to compare MPA prevalence in hospitalized adolescents suffering from AN to those with early onset psychosis and healthy controls. To the best of our knowledge, this is the first such study that is focused on MPA in eating disorders.

## 2 METHOD

The study was performed in accordance with the Declaration of Helsinki and was approved by the Ethics Committee at the Institute of Psychiatry and Neurology, Warsaw, Poland.

### 2.1 Participants

These consisted of all patients admitted to the Child and Adolescent Psychiatry Department of Institute of Psychiatry and Neurology either during 2013 or 2017–2019, who fulfilled the study criteria and whose legal guardians gave consent for taking part in the study. The time gap in the recruitment period was due to logistic difficulties at the time unrelated to our study procedures.

Inclusion criteria were:

SSD Group; diagnosed SSD, chapter F2 in ICD‐10
AN Group; diagnosed anorexia nervosa (AN), (codes F50.0 or F50.1 in ICD‐10), only in females (to avoid the marked gender imbalance due to the small number of hospitalized boys).

Exclusion criteria were a diagnosis of intellectual disability or a known disease associated with dysmorphic features.

There were 85 test participants analyzed: 41 patients diagnosed with SSD and 44 patients diagnosed with AN (Table 1). The study had a defined time‐frame into which all available patients were included therein.

**TABLE 1:** Demographic data and diagnoses breakdown

**Group**	**N**	**M:F, % females**	**Age (years), mean ± SD, range**	**Diagnoses breakdown**
SSD	41	13:28, 68% females	16.78 ± 1.03 (14.75–18.92)	Schizophrenia F 20 *n* = 34 Schizotypal disorder F21 *n* = 2 Acute and transient psychotic disorder F23 *n* = 1 Schizoaffective disorder F25 *n* = 1 Psychotic disorder, not specified F29 *n* = 3
AN	44	0:44, 100% females	16.19 ± 1.50 (13.0–19.75)	Anorexia nervosa F50.0 *n* = 41 Atypical anorexia nervosa F50.1 *n* = 3
HC	30	12:18, 66% females	16.34 ± 1.83 (13.0–19.08) Female subsample 16.38 ± 1.79 (13.0–19.08)	None

Abbreviations: SSD—schizophrenia spectrum disorders group, AN—anorexia nervosa group, and HC—healthy control group.

The control group consisted of 30 teenagers attending general school education. Exclusion criteria were having had psychiatric consultations at any time or if the subjects’ legal guardians had withheld their informed consent.

### 2.2 Procedure

#### 2.2.1 Diagnosis

Psychiatric diagnoses were established in a few steps procedure. A preliminary diagnosis was made by the attending clinician based on a psychiatric interview, (developed in house), with the patient and parents at admission. This interview was focused on patients’ history and clinical assessment according to ICD‐10 guidelines (WHO 1993). The second step was a psychiatric examination conducted by an experienced senior psychiatrist assisted by junior clinicians. A final diagnosis was made after further observation of symptoms during admission, which was then verified by a multidisciplinary treatment team.

#### 2.2.2 Study design

Diagnoses were as per above. During hospitalization, patients were graded according to the Waldrop MPA Scale. This is a ratings‐based tool consisting of a list of possible anomalies. The English version of the scale was used. Psychopathological symptoms were also assessed as described in another study (Remberk et al., 2018)

#### 2.2.3 Control group

This was assessed by the Waldrop Minor Physical Abnormalities Scale at the time when subjects were regularly attending their school office.

#### 2.2.4 Minor physical abnormalities assessment

The Waldrop Minor Physical Abnormalities Scale(Waldrop et al., 1968; Appendix 1) was used to assess MPA. This 17‐item scale lists those abnormalities localized on the head, mouth, hand and feet areas. To ensure reliability, each participant was assessed by the same two examiners (BR and PN). Anomalies were considered as being present if observed independently by the two examiners, otherwise they were taken as being absent. The total score and subscore for MPA localized in the head area were analyzed.

### 2.3 Statistical analysis

The variables’ distribution was tested for normality by the Kolmogorov‐Smirnov test (K‐S) and Shapiro‐Wilk (S‐W) test, where a non‐parametric distribution was indicated by both tests (*p* < 0.05). Statistical analyses were thus performed by the non‐parametric Mann–Whitney *U*‐test for between group comparison for ordinal variables and the chi‐squared test for categorical variables. The level of statistical significance was set as *p* < 0.05 while *p* < 0.08 was considered a statistical trend.

All statistical analyses were performed using SPSS software; Version 26.

## 3 RESULTS

There were no differences in age or gender distribution between the SSD group and controls. The AN group consisted only of females. There was also no difference in age between the AN and HC group nor between the AN group and female subsample of the HC group.

Demographic data and diagnoses breakdown are summarized in Table 1.

### 3.1 Minor physical abnormalities total score and head subscore

Statistically significant differences were found in mean MPA scores and in mean head subscores between the SSD and HC groups, between the AN and HC groups and between the AN and female HC subgroup. Higher MPA scores were observed in the patients groups compared to controls. There was no difference between SSD and AN groups. Data are summarized in Table 2.

**TABLE 2:** Total minor physical abnormalities score and head subscore

**Group**	**MPA total score median ± IQR,range**	**MPA Head subscore, median ± IQRrange**	***p***	***U***
SSD	1 ± 1,5 range 0–4	0.98 ± 0.91, 1 ± 1,5 range 0–3	SSD versus HC total score: *p* = 0.003 Head subscore: *p* = 0.03 SSD versus AN total score not significant Head subscore not significant	*U* = 355.5 *U* = 337 *U* = 891 *U* = 791
AN	1 ± 1 range 0–4	0.80 ± 0.93, 1 ± 1 range 0–4	AN versus HC total score: *p* = 0.003 Head subscore p = 0.02 AN versus female subsample of HC total score: *p* = 0.001 Head subscore *p* = 0.03	*U* = 370 *U* = 450 *U* = 170 *U* = 257
HC HC female subsample	0 ± 1 range 0–4 0 ± 1, range 0–2	0 ± 0 range 0–2 0 ± 0, range 0–1	SSD versus HC total score: *p* = 0.003 Head subscore: *p* = 0.03 AN versus HC total score: *p* = 0.003 Head subscore *p* = 0.02 AN versus female subsample of HC total score: *p* = 0.001 Head subscore *p* = 0.03	*U* = 355.5 *U* = 337 *U* = 370 *U* = 450 *U* = 170 *U* = 257

Abbreviations: SSD—schizophrenia spectrum disorders group, AN—anorexia nervosa group, HC—healthy controls group, and IQR—interquartile range

Rates of subjects without any MPA for SSD and AN groups were respectively 24% (*n* = 10) and 18% (*n* = 8), whereas they were significantly higher/improved in controls at 57% (*n* = 17). Likewise, there were significantly higher rates of whenever MPA anomalies were absent in the head region for controls 77% (*n* = 23), compared to those for the SSD and AN groups; respectively 34% (*n* = 14) and 45% (*n* = 20); Table 3.

**TABLE 3:** Subjects lacking minor physical abnormalities

**Group**	**MPA—none anomaly, *n* (%)**	***p***	**MPA—head area, none anomaly *n* (%)**	***p***
SSD	10 (24%)	SSD versus AN not significant SSD versus HC *p* = 0.006	14 (34%)	SSD versus AN not significant SSD versus HC *p* = 0.0004
AN	8 (18%)	AN versus HC *p* = 0.0006 AN versus female subsample of HC *p* = 0.0002	20 (45%)	AN versus HC *p* = 0.008 AN versus female subsample of HC *p* = 0.02
HC	17 (57%)	SSD versus HC *p* = 0.006 AN versus HC *p* = 0.0006	23 (77%)	SSD versus HC *p* = 0.0004 AN versus HC *p* = 0.008
HC female subsample	12 (66%)	AN versus female subsample of HC *p* = 0.0002	14 (78%)	AN versus female subsample of HC *p* = 0.02

Abbreviations: SSD—schizophrenia spectrum disorders group, AN—anorexia nervosa group, and HC—healthy controls group.

### 3.2 Minor physical abnormalities profile

**FIGURE 1:**
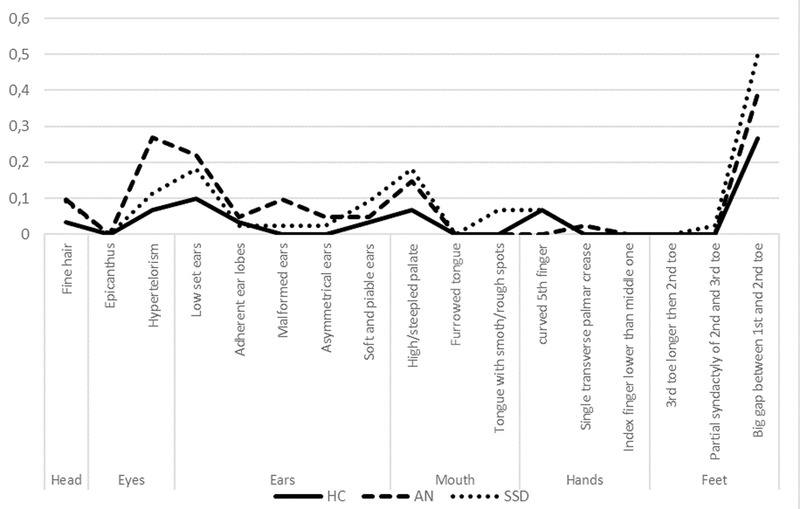
Percentage of patients with observed abnormalities. Acronyms: SSD—schizophrenia spectrum disorders group, AN—anorexia nervosa group, and HC—healthy control group

The most common anomalies found in the HC group were an enlarged gap between the first and second toe (*n* = 8, 27%) followed by low seated ears (*n* = 3, 10%). For the SSD group these were likewise an enlarged gap (*n* = 16, 39%) but followed next by hypertelorism (*n* = 11, 27%) and then by low seated ears (*n* = 9, 22%). In the AN group, there was an enlarged gap in 22 subjects (50%), followed by *n* = 8 (18%) low seated ears and also *n* = 8 (18%) gothic palate. The full MPA profiles are presented in Figure 1.

## 4 DISCUSSION

The presented study shows higher frequency of MPA in both patient test groups than in the HC. Such a high frequency in SSD subjects could be expected; in keeping with previous studies as summarized in the Introduction section.

To the best of our knowledge however, the presented study is the first of its kind to also focus on the association between MPA and AN. Indeed, we found a similar proportion of MPA in SSD and AN patients. The significantly higher frequency of MPA in AN patients than in HC is consistent with a suggested neurodevelopmental component in the etiology of AN.

Nevertheless, our sample group of inpatients may not be representative of the AN population as a whole.

Hospitalized patients usually have much more pronounced symptoms than those not admitted. Furthermore, severe disease courses are linked to poor insights about the disease and firmly held convictions. Konstantakopoulos et al. (2012) stated that the delusional variant of the disorder occurs at the end of the AN continuum. Indeed, psychotic symptoms have been observed in subjects suffering from eating disorders in some studies (Behar et al., 2018; Miotto et al. 2010).

Although the frequency of MPA was higher in both patient test groups, the profile of observed anomalies had similarities in both test groups and controls. This may indicate that MPA are not specific risk indicators of different psychiatric disorders. As aforementioned, MPA were not only observed in psychotic patients. Nevertheless, the association between MPA, schizophrenia and its increased genetic burden was confirmed (Hajnal et al., 2016; Wang et al., 2016). Similar profile of MPA found in HC and SSD groups is also in line with continuum model of psychosis. According to this concept, subclinical psychotic symptoms and psychotic‐like experiences (PLE) may be present in the general population which represents the lower end of continuum, whilst clinical cases lie at the upper end (van Os et al., 2009). Those of the general population with PLE, share some risk factors with schizophrenia subjects, however such risk factors are less prevalent in people without PLE (Tochigi et al., 2013; van Os et al., 2001).

Likewise, the range of AN symptoms in the general population may also be interpreted as a continuum, where subclinical difficulties are substantially prevalent in the general population at the lower end of the continuum, whilst the most severe delusional forms lie at the upper end.

It is instructive to consider our current findings within the context of studies focused on similarities between the disease courses and psychopathology of AN and psychotic disorders. Such issues include AN and psychosis comorbidity, where AN may be part of the premorbid phase of psychosis, whilst psychotic symptoms occur during the course of AN but without comorbidities; such findings are however not unequivocal (Morylowska‐Topolska et al., 2017).

Nevertheless, despite similarities in biological risk factors and some overlapping symptoms in clinical presentation, discrepancies between schizophrenia and AN are also substantial. Observed functional and neuroanatomical disturbances in these disorders are not equivalent (Mitelman, 2019). Similar MPA frequency and profile in both patients groups may also be interpreted as an indicator of low MPA specificity.

In summary, our findings are consistent with the neurodevelopmental model for both schizophrenia and AN. It should however be noted that further research is required on subjects not covered by our study, such as outpatients and males with AN, with focus on the association between neurodevelopmental indicators and subtypes of AN and MPA specificity for different psychiatric disorders.

### 4.1 Strengths and limitations of the study

A limitation of this study is a not very large sample size which is a common problem faced in child and adolescent psychiatry. Nevertheless, statistically significant results were obtained.

Any study of severe mental illnesses in adolescents will be inevitably limited by the fact that SSDs typically occur in early adulthood which is also a time when AN can be still quite frequent. Thus, some HC may anyway develop psychiatric disorders in the future, whilst diagnoses made on inpatients at this time may not constitute a lifetime diagnosis.

The Waldrop Scale does not have a Polish validated version. However, the scale is ratings‐based, thus we believe that English version has enabled us to perform a reliable assessment. Results were not compared to an external norm, but were used for making between groups comparisons. Moreover, previous studies have confirmed a universal, across‐ethnicity utility of the scale (Dean et al., 2007; Koen et al., 2006).

Choosing the appropriate statistical tests depends on the distribution of variables. Our study had to use non‐parametric statistics testing, which may have reduced the statistical power. This however only refers to there being no statistically significant differences between the AN and SSD groups. Nonetheless, our main findings demonstrate statistically significant differences between patients and controls which should not affected by this bias.

Our study only included inpatients, which may not be representative of the patient population as a whole; particularly for AN. Another drawback is the dearth of male anorexic subjects because of the very low numbers of hospitalized boys in our department. Nevertheless, AN in males needs to be investigated.

The strength of our study lies in the structured assessment procedure. We consider that the way MPA were assessed to be highly reliable due to having two independent examiners and ensuring that these same two are used for all subjects. An in‐depth assessment of patients was also ensured by the multi‐step diagnostic process that included a semi‐structured interview.

To the best of our knowledge this is the first study that has assessed MPA in patients with AN.

## 5 CONCLUSIONS

Available evidence indicates that MPA arises from processes acting during the early stages of embryonic and fetal existence. Our study outcomes demonstrate that such anomalies are over‐represented in those adolescents diagnosed with schizophrenia or AN, which thereby supports the notion that such disorders are related to pathological factors, (genetic and/or environmental), that occur in early development.

Our findings are consistent with the neurodevelopmental model for both schizophrenia and AN; however, the outcomes should not be overgeneralized and thus further investigations are warranted.

### PEER REVIEW

The peer review history for this article is available at https://publons.com/publon/10.1002/brb3.2281

## CONFLICT OF INTEREST

The authors declare no conflict of interest.

## Data Availability

The data that support the findings of this study are openly available in Mendeley data repository DOI: 10.17632/6cpp77s64r.1

## APPENDIX 1 Waldrop Minor Physical Abnormalities Scale

### 1.1

- Electric hair
  - Very fine hair that won't comb down
  - Fine hair that is soon awry after combing
  - Two or more whorls
- Epicantus
  - Where upper and lower lids join the nose, point of union is
  - Deeply covered
  - Partly covered
- Hypertylorism
- Approximate distance between tear ducts
  - = 1.5 inches
  - = 1.25 < 1.5
- Ears low seated
  - Bottom in ears in line with
  - Mouth or lower
  - Area between mouth and nose
- Adherent lobes
  - Lower edges of ears extend
  - Upward and back toward the crown of head
  - Straight back toward rear of neck
- Malformed ears
- Assymetrical ears
- Soft and pliable ears
- High palate
  - Definitely steepled
  - Flat and narrow at the top
- Furrowed tongue (one with deep ridges)
- Smooth‐rough spots on tongue
- Fifth finger
  - Markedly curved inward toward other fingers
  - Slightly curved inward toward other fingers
- Single transverse palmar crease
- Index finger lower than middle finger—0
- Third toe
  - Definitely longer than second toe—2
  - Appears equal in length to second toe—1
- Partial syndactylia of two middle toes
- Gap between first and second toe (approximately > = ¼ inch)
